# Minimally invasive prediction of blood lactate during incremental exercise via heart rate, core body temperature, and sweat-derived indices

**DOI:** 10.1038/s41598-026-47148-8

**Published:** 2026-04-07

**Authors:** Jaesung Lee, Jihye Moon, Youngim Kim, Hyeonmin Kim, Eunbi Kim, Hyunseob Lee, Sungjin Yoon, Seunghwan Chon, Youngkeun Lee, Jonghoon Park

**Affiliations:** 1 Department of Physical Education, Korea University, 145 Anam-Ro, Seongbuk-Gu, Seoul, 02841 Republic of Korea; 2 Department of Physical Education, Graduate School of Education, Korea University, Seoul, Republic of Korea; 3 Dongwoo Fine-Chem Co., Ltd. AR&D Center, 35, Poseunggongdan-ro 117 Beon-gil, Poseung-eup, Pyeongtaek-si, 17956 Gyeonggi-do Republic of Korea

**Keywords:** Blood lactate, Heart rate, Core body temperature, Sweat lactate, Metabolic monitoring, Wearable biosensor, Biomarkers, Cardiology, Health care, Medical research, Physiology

## Abstract

Blood lactate concentration (BLa) is a key marker of metabolic stress, but invasive sampling limits real-time monitoring. We developed a minimally invasive model to estimate BLa during incremental exercise using heart rate (HR), core body temperature (CBT), and sweat-derived indices. Thirty-one healthy adult males performed a graded treadmill test. HR and CBT were monitored continuously. Sweat was sampled from the forehead, chest, and back to quantify sweat lactate concentration ([La⁻]sw) and lactate excretion rate (LER = [La⁻﻿]sw × sweat rate). Linear mixed-effects models (LMMs) were fitted with log-transformed BLa (Log[BLa]) and participant-level random effects. BLa increased with exercise intensity (*p* < 0.001), accompanied by increases in HR, CBT and LER (both *p* < 0.001). LMMs combining HR, CBT, and sweat indices showed strong performance for Log[BLa]. The best model (HR + CBT+forehead LER) achieved conditional R²=0.939 and RMSE = 0.229 (log units), and forehead-based models outperformed chest and back. Combined cardiovascular, thermoregulatory, and sweat-derived measures enable accurate, minimally invasive estimation of BLa during graded exercise, supporting wearable-based metabolic monitoring and individualized exercise prescription.

**Supplementary Information:**

The online version contains supplementary material available at 10.1038/s41598-026-47148-8.

## Introduction

Blood lactate concentration (BLa) increases with exercise intensity and serves as a key indicator of aerobic capacity and endurance performance; accordingly, it is widely used in sports science and clinical rehabilitation^1,2^. However, conventional BLa assessment during exercise requires invasive blood sampling, which limits continuous and real-time monitoring due to discomfort, procedural complexity, and potential infection risk^3^.

Recent advances in wearable devices for sweat lactate measurement have enabled non-invasive approaches to estimating BLa^4–7^. In addition studies on wearable and microfluidic sweat lactate sensing have reported associations between sweat-derived lactate signals and exercise intensity, suggesting their potential usefulness for continuous or prolonged monitoring during exercise^8–10^. Importantly, sweat lactate concentration ([La⁻]sw) does not reflect passive diffusion from blood. It can also be locally produced through glycolytic activity within sweat gland cells, and is influenced by multiple physiological and environmental factors, including sweat rate, skin blood flow, and thermal stress^11,12^. As sweat rate increases, dilution effects may attenuate the association between [La⁻]sw and BLa^12^. To partially account for dilution, lactate excretion rate (LER; [La⁻]sw × sweat rate) has been proposed as a more informative index^13^. Nevertheless, regional differences in sweat gland density and sweating responses, as well as variation in sensor attachment and skin–sensor contact, may limit the representativeness of a single local measurement^14^. This suggests that additional physiological variables may be necessary to improve the robustness of non-invasive BLa estimation.

Heart rate (HR) and core body temperature (CBT) are potential physiological signals that may reflect metabolic strain beyond cardiovascular or sweat-derived indices^15,16^. HR typically increases with exercise intensity and shows moderate-to-strong associations with BLa, supporting its use for lactate estimation^15^. However, inter-individual variability in cardiovascular responses, training status, and fatigue resistance can reduce predictive reliability^16^. CBT also rises progressively during exercise due to metabolic heat accumulation, and elevations in body temperature have been linked to increased glycolytic activation and lactate accumulation^17,18^. Despite this physiological rationale, CBT has rarely been incorporated as a predictor of lactate dynamics, as prior work has primarily focused on thermoregulatory function or heat-related illness risk^19^. Although handheld lactate testing is practical and field-deployable, it remains limited to intermittent point measurements and does not capture the continuous dynamics of blood lactate during exercise. Therefore, integrating HR, CBT, and sweat-derived lactate indices may provide a more robust approach for closer-to-real-time monitoring of metabolic trends, particularly in settings where repeated blood sampling is impractical.

Accordingly, the aim of this study was to develop and validate multivariable models for predicting BLa during incremental exercise using physiological signals that could reduce reliance on repeated blood sampling. We constructed regression models incorporating HR, CBT, and sweat-derived lactate indices. Based on the optimal combination of explanatory variables identified using a linear mixed-effects model, we additionally applied a random forest algorithm using the same inputs to evaluate predictive performance.

## Results

### Stage-dependent responses of physiological variables

**Fig. 1:**
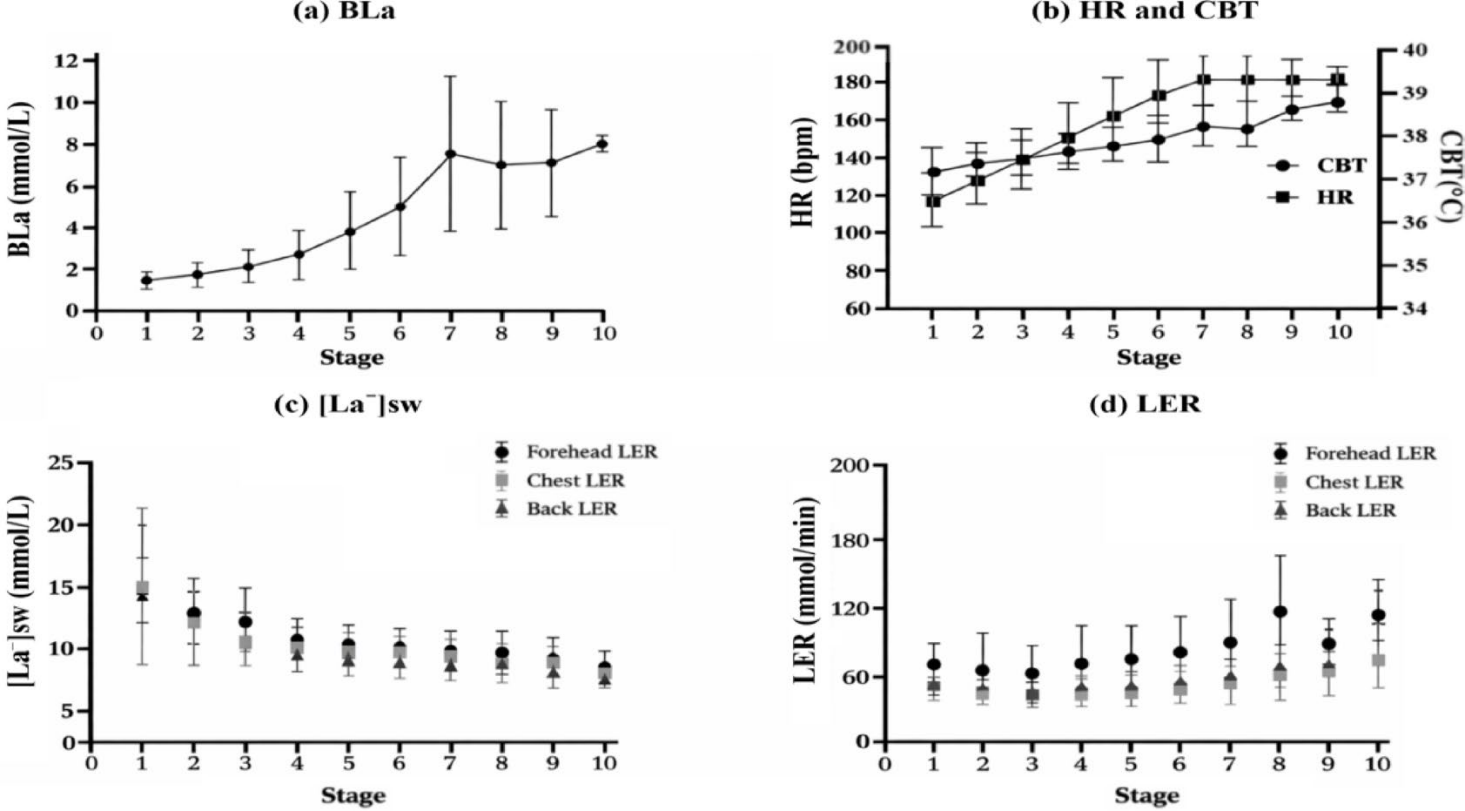
Changes in blood lactate concentration (BLa), heart rate (HR), core body temperature (CBT), sweat lactate concentration ([La⁻]sw), and lactate excretion rate (LER) during incremental exercise. **(a)** BLa, **(b)** HR and CBT, **(c)** [La⁻]sw, and **(d)** LER responses across exercise stages. Data are presented as mean ± SD. Abbreviations: BLa, blood lactate concentration; HR, heart rate; CBT, core body temperature; [La⁻]sw, sweat lactate concentration; LER, lactate excretion rate.

As shown in Fig. 1a, blood lactate concentration (BLa) increased progressively with increasing exercise intensity, showing marked elevation from stage 4 and peaking at stage 7 (*p* < 0.001). Heart rate (HR) and core body temperature (CBT) significantly increased with workload (*p* < 0.001; Fig. 1b), with HR increasing steadily from ~ 100 bpm to ~ 180 bpm and plateauing near stage 7, and CBT rising sharply between stages 6 and 8 to ~ 39 °C. Lactate excretion rate (LER) at the forehead, chest, and back significantly increased with exercise intensity (*p* < 0.001; Fig. 1d), with the forehead showing the largest rise before stabilizing at higher stages. In contrast, sweat lactate concentration ([La⁻]sw) significantly decreased across stages (*p* < 0.001; Fig. 1c), peaking at stage 0 and a progressive decline that stabilized from stage 5 onward.

### Correlations between BLa and physiological variables

**Fig. 2:**
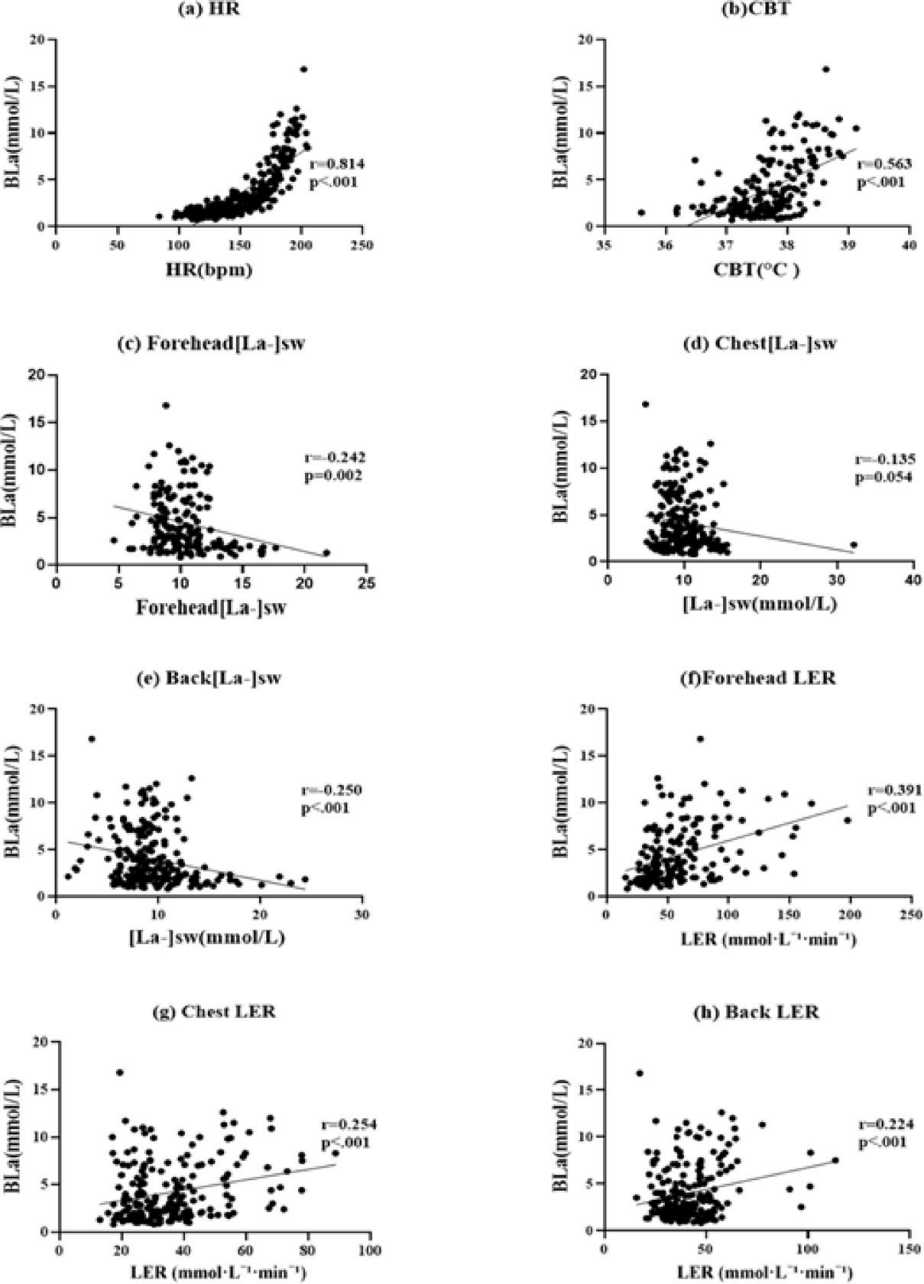
Correlations between blood lactate concentration (BLa) and physiological variables during incremental exercise. **(a)** HR, **(b)** CBT, **(c)** forehead [La⁻]sw, **(d)** chest [La⁻]sw, **(e)** back [La⁻]sw, **(f)** forehead LER, **(g)** chest LER, and **(H)** back LER. Abbreviations: BLa, blood lactate concentration; HR, heart rate; CBT, core body temperature; [La⁻]sw, sweat lactate concentration; LER, lactate excretion rate.

Figure 2 shows the correlations between BLa and physiological variables. HR strongly correlated with BLa (*r* = 0.814, *p* < 0.001; Fig. 2a), and CBT showed a moderate positive correlation (*r* = 0.563, *p* < 0.001; Fig. 2b). [La⁻]sw exhibited weak negative correlations across measurement sites—forehead (*r* = − 0.242, *p* = 0.002; Fig. 2c) and back (*r* = − 0.250, *p* < 0.001; Fig. 2e). LER showed significant positive correlations with BLa—forehead (*r* = 0.391, *p* < 0.001; Fig. 2f), chest (*r* = 0.254, *p* < 0.001; Fig. 2g), and back (*r* = 0.224, *p* < 0.001; Fig. 2h).

Predictor intercorrelations were generally low to moderate, although stronger associations were observed between regional sweat-derived indices, particularly between [La⁻]sw and LER measured at the same site (*r* = 0.764–0.796). HR and CBT showed a moderate correlation (*r* = 0.514). However, multicollinearity in the final multivariable models was low, with all VIF values ranging from 1.04 to 1.48 (Supplementary Table S1).

### Regression models for predicting blood lactate concentration

Table 1 summarizes the regression performance for predicting BLa using various physiological signal combinations. Conditional and marginal R² values and corresponding RMSE were used to evaluate each model’s explanatory power and prediction accuracy. Models that included HR demonstrated the highest predictive capability, with conditional R² values consistently > 0.90. Among these, combining HR, CBT, and forehead LER yielded the best performance (conditional R² = 0.939, marginal R² = 0.788, conditional RMSE = 0.2288). Models using CBT alone showed moderate predictive accuracy (conditional R² = 0.799, marginal R² = 0.481). Predictive performance improved when CBT was combined with sweat-derived parameters such as LER or [La⁻]sw. The CBT + forehead LER model achieved a conditional R² of 0.901 and a conditional RMSE of 0.3662. Overall, regression models integrating cardiovascular (HR) and thermoregulatory (CBT) variables with sweat-derived parameters (LER or [La⁻]sw) exhibited conditional R² values ranging from 0.921 to 0.939. Among the multivariable models, the combination of HR, CBT, and forehead LER showed the best overall predictive performance. Across the multivariable models, HR generally contributed most strongly to prediction, while CBT and sweat-derived indices provided additional explanatory value when combined with HR. All final multivariable regression models were statistically significant (all *p* < 0.001; Supplementary Table S2). Based on the standardized regression coefficients, HR showed the largest contribution across models, whereas CBT showed moderate contributions and sweat-derived indices showed smaller but consistently positive contributions to BLa estimation (Supplementary Table S2, S3). When model fit was additionally evaluated using AIC, the HR + CBT + Forehead [La⁻]sw model showed the lowest AIC among the candidate models, indicating the most favorable balance between model fit and model complexity. Residual diagnostics and formal assumption checks were additionally performed for the candidate regression models. Although some models showed minor deviations in individual diagnostic tests, no major convergence or singular-fit problems were observed, and the models were considered acceptable for interpretation (Supplementary Table S4). As a supplementary internal validation, random forest models were applied using the same predictor set with a 70:30 train–test split. Test-phase R² values ranged from 0.690 to 0.807 and test RMSE values ranged from 1.224 to 1.492 mmol·L⁻¹, supporting the robustness of the selected multi-signal variables under a nonlinear framework.

**Table 1:** Regression performance (R², RMSE) by physiological signal combination.

Factor	Conditional *R*²	Marginal *R*²	Conditional RMSE	Marginal RMSE
HR	0.908***	0.795***	0.2973	0.4678
HR + CBT	0.917***	0.807***	0.2818	0.4554
HR+Forehead[La⁻]sw	0.926***	0.807***	0.2648	0.4618
HR+Chest[La⁻]sw	0.918***	0.808***	0.2795	0.4510
HR+Back[La⁻]sw	0.916***	0.791***	0.2839	0.4681
HR+Forehead LER	0.921***	0.785***	0.2596	0.4561
HR+Chest LER	0.915***	0.797***	0.2779	0.4556
HR+Back LER	0.912***	0.789***	0.2829	0.4614
HR + CBT+Forehead[La⁻]sw	0.933***	0.818***	0.2429	0.4248
HR + CBT+Chest[La⁻]sw	0.921***	0.828***	0.2698	0.4231
HR + CBT+Back[La⁻]sw	0.926***	0.799***	0.2675	0.4634
HR + CBT+Forehead LER	0.939***	0.788***	0.2288	0.4572
HR + CBT+Chest LER	0.921***	0.806***	0.2684	0.4502
HR + CBT+Back LER	0.933***	0.792***	0.2668	0.4671
CBT	0.799***	0.481***	0.5549	0.9517
CBT+Forehead[La⁻]sw	0.871***	0.460***	0.4283	0.9407
CBT+Chest[La⁻]sw	0.854***	0.503***	0.4691	0.9873
CBT+Back[La⁻]sw	0.838***	0.522***	0.4816	0.9244
CBT+Forehead LER	0.901***	0.512***	0.3662	0.8807
CBT+Chest LER	0.854***	0.503***	0.4679	0.9804
CBT+Back LER	0.877***	0.505***	0.4507	1.0101

^***^p < 0.001, Note: RMSE values are reported on the log-transformed BLa scale (log units).

### Agreement between predicted and observed BLa

**Fig. 3:**
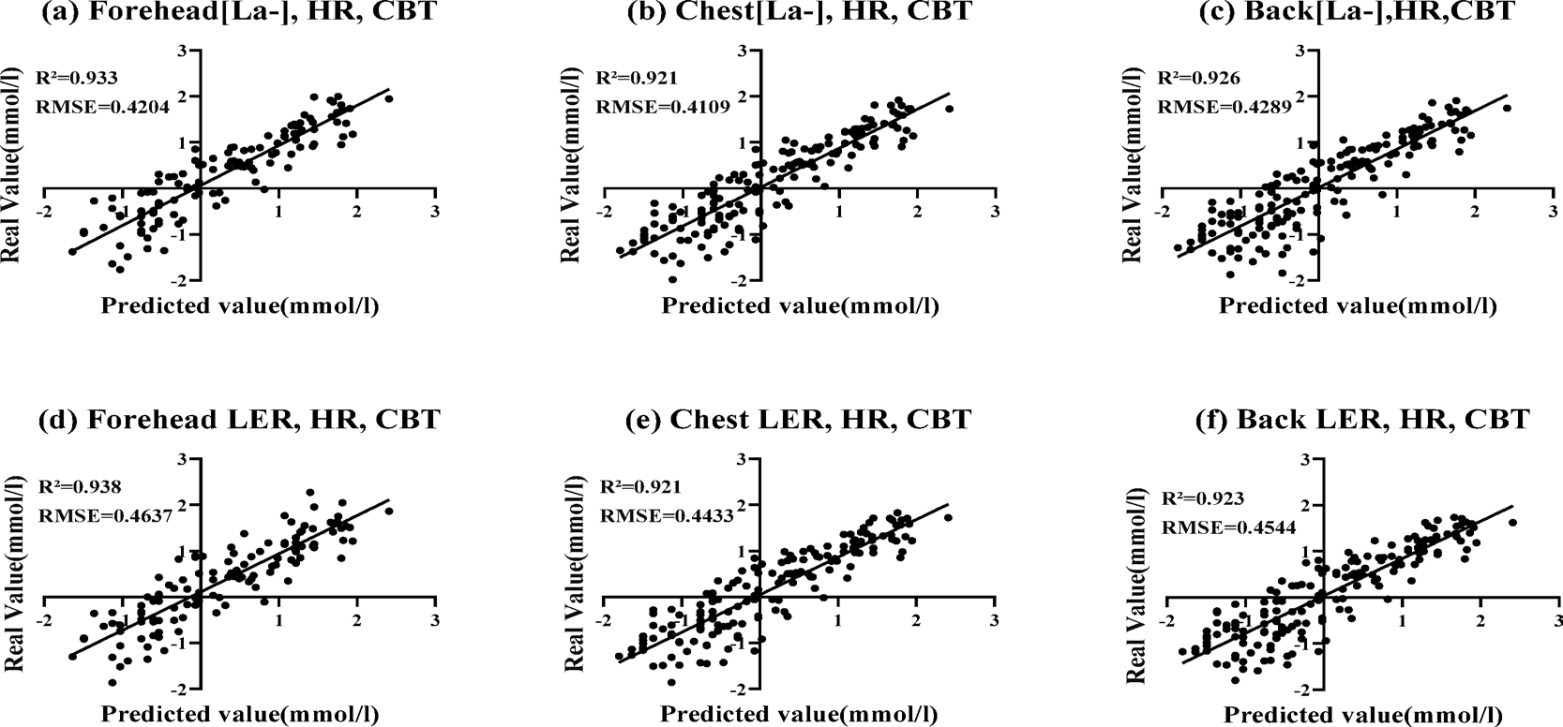
Agreement between predicted and observed log-transformed blood lactate concentration (log[BLa]) across body regions based on conditional predictions from the linear mixed-effects model (LMM). **(a)** Forehead [La⁻]sw + HR + CBT, **(b)** Chest [La⁻]sw + HR + CBT, **(c)** Back [La⁻]sw + HR + CBT, **(d)** Forehead LER + HR + CBT, **(e)** Chest LER + HR + CBT, and **(f)** Back LER + HR + CBT. All input variables (HR, CBT, and sweat-derived lactate indices) were standardized (z-scores) to account for scale differences and multicollinearity. Abbreviations: BLa, blood lactate concentration; HR, heart rate; CBT, core body temperature; [La⁻]sw, sweat lactate concentration; LER, lactate excretion rate; LMM, linear mixed-effects model.

Figure 3 presents the agreement between predicted and observed log-transformed blood lactate concentration (Log[BLa]) values across different body regions, based on conditional predictions from the linear mixed-effects model (LMM). Log-scale values can be back-transformed to the original scale using the exponential function (BLa = exp(Log[BLa])). Therefore, differences on the log scale correspond to multiplicative (ratio) differences in BLa. Predictive models incorporating HR, CBT, and sweat-derived variables showed strong consistency between predicted and measured values. Among [La⁻]sw-based models, combining forehead [La⁻]sw, HR, and CBT yielded the highest predictive accuracy (R² = 0.933, RMSE = 0.4204; Fig. 3a), followed by chest [La⁻]sw (R² = 0.921, RMSE = 0.4109; Fig. 3b) and back [La⁻]sw (R² = 0.926, RMSE = 0.4289; Fig. 3c). When LER was used instead of concentration, similar predictive performance was observed. The forehead LER model showed the strongest agreement (R² = 0.938, RMSE = 0.4637; Fig. 3d). The chest and back LER models also exhibited high predictive accuracy (R² = 0.921, RMSE = 0.4432; Fig. 3e; R² = 0.923, RMSE = 0.4544; Fig. 3f). The LMM models achieved high predictive accuracy for Log(BLa) across all body regions, indicating consistently strong relative agreement in (multiplicative) terms after back-transformation to the original BLa scale.

## Discussion

This study demonstrated that blood lactate concentration during graded exercise can be reliably estimated by integrating physiological signals. Specifically, HR and CBT exhibited strong and moderate positive correlations with BLa, whereas the lactate excretion rate (LER) showed a significant yet modest correlation. Regression analyses revealed that combined models incorporating HR, CBT, and sweat-derived parameters—particularly forehead LER—achieved the highest predictive accuracy, with conditional R² values > 0.93. Linear mixed-effects modeling enhanced interpretability and stability of predictions across participants. Collectively, these findings highlight the feasibility of integrated cardiovascular, thermoregulatory, and sweat-based indices for monitoring metabolic stress without repeated blood sampling, with applications in exercise testing, performance optimization, and clinical rehabilitation.

In this study, all measured physiological variables exhibited characteristic response patterns across progressive exercise stages, reflecting coordinated cardiovascular, thermoregulatory, and metabolic system activation.

BLa increased progressively with exercise intensity, consistent with established lactate accumulation patterns during incremental workloads^1^. HR rose in a near‑linear manner and plateaued near maximal exertion (Fig. 1), which reflects the expected cardiovascular response to graded exercise and aligns with previous reports showing steady increases in HR before leveling off at high intensities^8,17^. CBT increased continuously across stages, consistent with the progressive accumulation of metabolic heat and the imbalance between heat production and dissipation during sustained exercise^9,12^. In contrast, [La⁻]sw decreased with increasing workload, whereas LER increased. This divergence is physiologically plausible: higher sweat rates at greater intensities can dilute sweat lactate concentration while simultaneously increasing total lactate excretion^11,12^. The overall trends in Fig. 1 likely reflect rising metabolic demand, enhanced sweating responses, and a time lag between blood lactate and sweat‑derived indices^1,11,12^. Recent sweat lactate sensing studies also report similar exercise‑related changes in sweat‑derived lactate signals^8,9^. Taken together, these variable-specific responses indicate that the overall trends observed in Fig. 1 were physiologically plausible and broadly consistent with expected cardiovascular, thermoregulatory, and sweat-related adaptations to incremental exercise.

Correlation analyses were conducted to evaluate the physiological relationships among these variables during exercise. HR showed a strong positive correlation with BLa (*r* = 0.814, *P* < 0.001), consistent with prior evidence linking HR to metabolic intensity and lactate kinetics during graded exercise^10,15^. CBT demonstrated a moderate positive association with BLa (*r* = 0.563, *P* < 0.001), aligning with previous findings that elevated internal temperature parallels increased lactate accumulation owing to enhanced glycolytic flux under thermal load^13,17^. The weaker correlation of CBT relative to HR likely reflects the multifactorial nature of thermoregulation, which is influenced by metabolic heat production, heat dissipation mechanisms such as sweating, skin blood flow, and environmental conditions^20,21^. Prior studies have similarly reported progressive increases in CBT with workload; however, it shows greater variability due to environmental and individual thermoregulatory differences, weakening its direct association with lactate kinetics^19,22^.

[La⁻]sw exhibited the weakest negative correlations with BLa (–0.135 ≤ *r* ≤–0.250) among the measured variables in this study. This weak association reflects the strong influence of local factors on [La⁻]sw rather than systemic metabolic status. Specifically, sweat composition depends on sweat gland activity, skin blood flow, evaporation rate, and local dilution effects, which vary across body sites and individuals. With increasing exercise intensity, total lactate excretion increases; however, sweat concentration decreases owing to higher sweat volume and dilution, obscuring its direct relationship with blood lactate^8,9^. Regional variability (forehead vs. chest vs. back) and temporal lag between sweat and blood lactate changes further reduce the predictive value of sweat lactate concentration^23^.

Regression analyses further demonstrated that models combining HR, CBT, and sweat-derived parameters yielded the most accurate predictions of BLa (Table 1). Conditional and marginal R² values and RMSE showed that integrated models consistently outperformed single-variable approaches, consistent with recent findings emphasizing multi-signal integration for physiological modeling^5^. The model combining HR, CBT, and forehead LER achieved the best performance (conditional R² = 0.939; marginal R² = 0.788; RMSE = 0.2288). This finding is broadly consistent with previous studies suggesting that the forehead is a practical site for exercise sweat sensing because of its high sweat availability and suitability for sensor placement during exercise^24–26^. In the present study, these characteristics may have contributed to the better predictive performance of forehead-derived signals. However, this result should be interpreted cautiously, as regional sweat patterns can vary across individuals and exercise conditions, and the upper back has also been identified as a high-sweat region^24^. In addition, models pairing CBT with sweat-derived variables improved prediction accuracy compared with CBT alone, suggesting that thermal and sweat-derived markers provide complementary information for lactate estimation.

Log transformation of lactate data and application of the linear mixed-effects model (LMM) improved model fit and interpretability, as recommended for physiological datasets exhibiting non-linear accumulation patterns^27^. The LMM incorporated both fixed (HR, CBT, and sweat-derived indices) and random effects (participant variability), enabling accurate estimation of within-subject trends. Although results are presented on the log scale, model effects can be interpreted on the original BLa scale via back-transformation; specifically, exp(β) denotes a multiplicative change (ratio) in BLa for a one-unit change in the predictor, and log-scale predicted values can be converted to BLa using BLa = exp(Log[BLa]). Conditional R² values across all body regions ranged from 0.921 to 0.939, indicating consistent predictive accuracy between predicted and observed log-transformed BLa values. This important methodological approach improves model stability and interpretability, particularly during high-intensity exercise stages.

Because CBT was measured using an ingestible telemetric capsule, the present framework should be regarded as minimally invasive rather than fully non-invasive. These findings support the potential feasibility of the proposed multimodal framework for tracking exercise-related metabolic trends when repeated blood lactate sampling is impractical. However, further validation is required before broader real-world application. Despite these promising findings, this study had several limitations. First, the study included only healthy young adult males, which limits generalizability. Because sex-related physiological differences may influence lactate- and sweat-derived measures, the present findings should not be assumed to apply to females. Future studies should validate this framework in women and more diverse populations. Second, manual and intermittent sweat sampling was performed may have limited temporal resolution. The use of wearable, real-time sweat biosensors could improve capture of dynamic lactate fluctuations with higher fidelity. Third, although HR, CBT, and sweat-derived parameters captured key physiological domains, integrating additional signals—such as skin temperature response—may further enhance prediction accuracy.

Collectively, these findings demonstrated that integrating minimally invasive physiological signals—specifically HR, CBT, and sweat-derived lactate measures—provides a robust and physiologically interpretable framework for estimating BLa during graded exercise. The strong agreement between predicted and observed outcomes supports the potential applicability of this method for closer-to-real-time metabolic monitoring without repeated blood sampling, with potential implications for individualized exercise testing, performance optimization, and clinical rehabilitation.

## Methods

### Participants

Thirty-one healthy adult males aged 20–39 years were recruited. To minimize physiological variability and clearly examine the fundamental relationships among HR, CBT, sweat lactate, and blood lactate, this initial study intentionally recruited a homogeneous male sample. Sex‑related differences in body composition, sweating responses, thermoregulation, and hormonal fluctuations can introduce additional variability in lactate‑ and sweat‑based measures^28–30^; therefore, only male participants were included for this initial model validation. All participants engaged in ≥ 150 min of exercise per week for ≥ 3 months and maintained stable body weight (± 10%) during the previous 6 months. This criterion was applied to reduce potential confounding from recent changes in energy balance, body composition, and hydration status, which may influence thermoregulatory strain, sweating responses, and exercise-related physiological outcomes^31–33^. Table 2 presents the physical characteristics of participants (*n* = 31). None used medication and all complied with a 12‑hour fast before testing. Exclusion criteria included metabolic, cardiovascular, or neurological disorders; Type 1 or Type 2 diabetes; orthopedic limitations; history of surgery; or non-obesity related cancer. This study was approved by the Institutional Review Board of Korea University (KUIRB‑2024‑0280‑01), and written informed consent was obtained from all participants. All methods were carried out in accordance with relevant guidelines and regulations and the Declaration of Helsinki.

**Table 2:** Descriptive characteristics of participants (*n* = 31).

	Mean ± SD	Minimum	Maximum
**Age(yr)**	26.2 ± 5.82	18	37
**Height(cm)**	176 ± 4.96	167	188
**Weight(kg)**	73.4 ± 9.69	58	99.9
**Body Fat(%)**	14.7 ± 4.5	6.5	23.3

### Experimental procedure

Participants visited the laboratory on two occasions. The indoor environment was maintained at 29–31 °C and 50–70% relative humidity. At visit 1, written informed consent was obtained, followed by a preliminary assessment to confirm adequate sweating capacity. Body composition was assessed using a stadiometer and bioelectrical impedance analysis (InBody 270, Biospace, Seoul, Korea). While wearing a heart-rate monitor (Polar 810i, Polar Electro Oy, Finland), participants performed 30 min of treadmill exercise at 60% intensity based on previous protocols^7,13^. Using a gravimetric method^34^, participants with a local sweat rate (LSR) ≤ 0.4 mg/cm2/min were excluded. For visit 2, participants were instructed to refrain from alcohol consumption, strenuous exercise, and smoking for at least 24 h before testing. They also fasted for ≥ 12 h, avoided caffeine-containing beverages, and maintained adequate hydration. These procedures were implemented to standardize physiological conditions and minimize potential confounding factors. Participants consumed the telemetric core-body temperature capsule with water approximately 2 h before testing. A licensed nurse collected venous blood samples immediately after each exercise stage. Thereafter, sweat samples were collected using double-film absorbent patches placed on the forehead, chest, and upper back.

### Exercise protocol

**Fig. 4:**
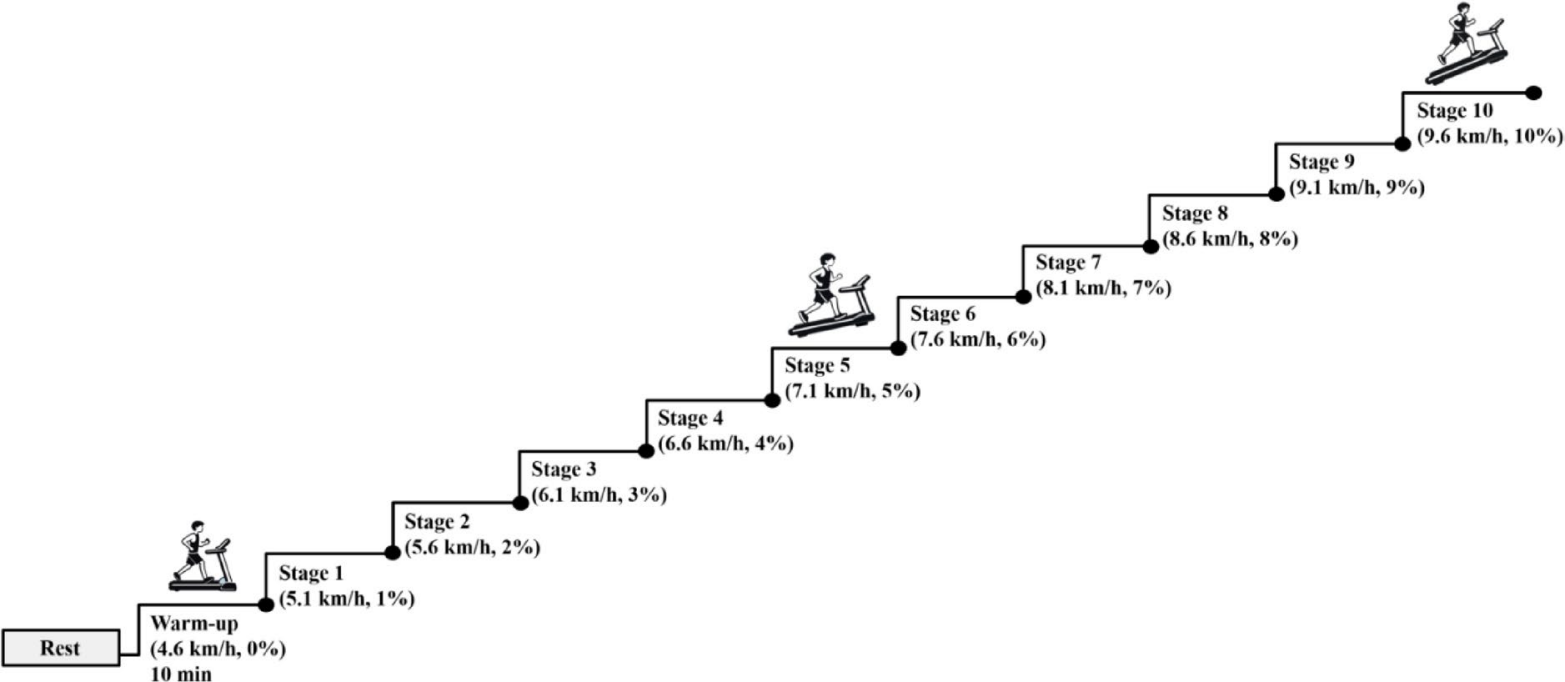
Experimental protocol and stage definitions for the graded treadmill test. Following baseline rest and a 10-min warm-up at 4.6 km/h and 0% incline, participants performed a graded treadmill test with progressive increases in speed and incline across stages. Each stage lasted 5 min and was followed by a 2-min rest period during which HR, CBT, blood lactate, and sweat samples were collected. Speed increased by 0.5 km/h and incline by 1% at each stage. The test continued until volitional exhaustion.

The exercise test was performed on a motorized treadmill (Drex NR20 NR20X, Drex, Korea) according to a modified Bruce protocol (Fig. 4). The test began with a 10-minute warm-up phase at 4.6 km/h and 0% incline, followed by a 2-minute rest period. The modified Bruce protocol was adopted to secure sufficient sweat volume over progressive workloads, enabling reliable sampling across both pre- and post-lactate threshold stages. This gradual intensity progression ensured stable thermoregulatory activation, facilitating continuous sweat excretion while delaying abrupt lactate accumulation. The protocol was suitable for this study because sustained sweat availability was essential for analyzing sweat-derived lactate biomarkers throughout dynamic metabolic transitions. The incremental treadmill test subsequently commenced, consisting of 5-minute stages separated by 2-minute rest periods. Speed increased by 0.5 km/h and incline by 1% at each stage, as detailed in Fig. 4. The test continued until volitional exhaustion, defined as the inability to maintain the required pace despite verbal encouragement.

### Blood sampling and analysis

A 22 G catheter (Kovax-Cath, Korea) was inserted into the antecubital vein of the non-dominant arm before exercise. Venous blood samples (~ 3 mL) were collected immediately after each exercise stage. Samples were stored at 4 °C and analyzed colorimetrically for blood lactate concentration at a certified clinical laboratory (Seegene Medical Foundation, Seoul, Korea).

## Measurements

### Heart rate (HR)

HR was continuously monitored using a chest-worn heart rate sensor. Resting HR was recorded during a seated rest period before exercise. HR values were recorded 10 s before each stage ended.

### Sweat sampling and analysis

Sweat collection patches were applied after the warm-up phase, after skin cleansing with 70% isopropyl alcohol and deionized water to remove debris and oils. To minimize interference from clothing contact and heat retention during sweat collection, upper-body garments were removed during testing. Sweat samples were collected continuously using double-film patches placed on the forehead (midline, 1 cm above the eyebrows), chest (lateral side, near the pectoral muscle), and upper back (most medial side of the scapula), based on an anatomical mapping method^24^. These sites were selected based on practical and physiological considerations, including relatively stable patch attachment during exercise, sufficient sweat availability for reliable signal acquisition, and prior use in regional sweat mapping and wearable sweat-sensing studies^24–26^. Each patch consisted of an absorbent cotton layer, a parafilm barrier (Disposable consumable laboratory film, CNWTC, Chongqing, China), and a transparent waterproof dressing (Tegaderm Film, 3 M, St. Paul, MN, USA). Cotton sizes were 16.8, 33.6, and 33.6 cm² for the forehead, chest, and back, respectively, with corresponding parafilm sizes of 19.2, 38.2, and 38.4 cm. Patches were secured during each 5-minute exercise stage and replaced afterward. Only sweat patches producing ≥ 2 µL of sweat were analyzed to ensure adequate sample size. Because sweat lactate was included as a predictor variable, sufficient sweat volume and analyzable signal quality were required for inclusion. Individuals with minimal sweating or delayed sweat onset could not provide reliable sweat lactate data, particularly during lower‑intensity stages, where sweat volume may be insufficient for accurate quantification^5,35^. Collected sweat samples were immediately weighed using an analytical balance (Hansung HS-103, Hansung Instrument, South Korea; precision: 0.001 g) to determine sweat volume, and subsequently centrifuged at 2,000 rpm for 5 min at 4 °C (LC-8 S, JoanLab, China) to separate the supernatant for further analysis. Samples were stored at 4 °C prior to analysis. [La⁻]sw was quantified amperometrically at 0.1 V with a calibrated lactate sensor (Dongwoo Fine-chem Inc.) connected to a CHI-630 electrochemical workstation (CH Instruments, Inc.). The sensor was calibrated daily with standard lactate solutions (0–20 mM) to ensure measurement accuracy within ± 5%.

### Core body temperature (CBT)

CBT was continuously monitored using an ingestible telemetric capsule transmitting data wirelessly to an external receiver (e-Viewer^®^ Performance). This method has been widely used to assess gastrointestinal temperature as a surrogate of core body temperature during exercise^36^. Data were recorded throughout the test and recovery period to assess thermoregulatory and metabolic responses.

### Statistical analyses

Statistical analyses were performed using Jamovi version 2.3 for Mac (The Jamovi Project, Sydney, Australia). Statistical significance was set at α = 0.05, data are presented as the mean ± standard deviation (SD) unless otherwise stated. Linear mixed-effects models (LMMs) were used to assess associations between blood lactate concentration (BLa) and physiological variables (HR, CBT, [La⁻]sw, and LER). BLa data were log-transformed to improve normality, and participant-level random effects accounted for repeated measures across exercise stages. Pearson’s correlation analyses additionally assessed linear relationships between BLa and each physiological variable. Intercorrelations among candidate predictors (HR, CBT, [La⁻]sw, and LER across body regions) were also examined using Pearson correlation coefficients, and multicollinearity in the final regression models was evaluated using variance inflation factors (VIFs). Model assumptions were assessed by inspection of residuals-versus-fitted plots for linearity, Q–Q plots and Shapiro–Wilk tests for residual normality, and Breusch–Pagan tests for homoscedasticity. Model convergence and singular fit were also checked for all candidate models. To confirm the robustness of the LMM findings, a supplementary random forest regression was applied using the same input variables (HR, CBT, and sweat-derived lactate variables) to explore the predictive relationships under a non-parametric framework. For this supplementary analysis, the dataset was randomly divided into training and test sets in a 70:30 ratio for internal evaluation of predictive performance. This analysis served as a secondary reference, suggesting that the variable combination retained predictive relevance beyond linear assumptions.

## Perspective

Increasing interest in wearable biosensors has renewed the need for valid indicators of metabolic strain during exercise that reduce reliance on repeated blood sampling. Our findings show that integrating HR and CBT with sweat-derived lactate indices, particularly forehead LER, enables accurate estimation of log-transformed blood lactate during incremental running. This supports the view that multi-signal monitoring can outperform single-marker approaches when lactate dynamics are influenced by cardiovascular, thermoregulatory, and sweat-gland factors. Practically, these models may facilitate closer-to-real-time intensity profiling and individualized training prescription without repeated blood sampling. Future studies should validate performance across sex, fitness levels, environments (heat/humidity), and exercise modes, and test device-level implementation with prospective external validation and error thresholds that are meaningful for coaching and clinical decision-making.

## Supplementary Information

Below is the link to the electronic supplementary material.

Supplementary Material 1

## Data Availability

The data supporting the findings of this study, including the raw and processed data underlying the figures and tables, are available from the corresponding author upon reasonable request.
