# Supplementary material for: Minimally invasive prediction of blood lactate during incremental exercise via heart rate, core body temperature, and sweat-derived indices
Source: Sci Rep. 2026 Apr 7;16:16547. doi: 10.1038/s41598-026-47148-8 (PMC13216596; doi:10.1038/s41598-026-47148-8)
Supplement: Supplementary file 1 — Supplementary Material 1 [file 41598_2026_47148_MOESM1_ESM.pdf]

Supplementary Table

Supplementary Table S1. Variance inflation factors (VIFs) and tolerance values for final multivariable regression models.

Model	Predictor	VIF	Tolerance
HR + CBT + Forehead [La$^-$]sw	HR	1.37	0.730
HR + CBT + Forehead [La$^-$]sw	CBT	1.29	0.777
HR + CBT + Forehead [La$^-$]sw	Forehead [La$^-$]sw	1.13	0.887
HR + CBT + Chest [La$^-$]sw	HR	1.35	0.740
HR + CBT + Chest [La$^-$]sw	CBT	1.30	0.768
HR + CBT + Chest [La$^-$]sw	Chest [La$^-$]sw	1.04	0.958
HR + CBT + Back [La$^-$]sw	HR	1.40	0.715
HR + CBT + Back [La$^-$]sw	CBT	1.33	0.749
HR + CBT + Back [La$^-$]sw	Back [La$^-$]sw	1.06	0.944
HR + CBT + Forehead LER	HR	1.27	0.790
HR + CBT + Forehead LER	CBT	1.34	0.745
HR + CBT + Forehead LER	Forehead LER	1.13	0.887
HR + CBT + Chest LER	HR	1.39	0.719
HR + CBT + Chest LER	CBT	1.30	0.767
HR + CBT + Chest LER	Chest LER	1.11	0.903
HR + CBT + Back LER	HR	1.48	0.674
HR + CBT + Back LER	CBT	1.32	0.755
HR + CBT + Back LER	Back LER	1.18	0.848

VIF was calculated from fixed-effects equivalent linear models using the same analytic dataset and predictor set as the corresponding mixed-effects models. All VIF values were below 5, indicating no evidence of problematic multicollinearity.

Supplementary Table S2. Standardized regression equations for estimating blood lactate concentration using HR, CBT, and sweat-derived lactate indices across body regions.

Body Region	Factor	Regression equation
Forehead	$[La^-]sw$	BLa = -0.0150 + 1.0090·HR + 0.1580·CBT + 0.1142·Forehead$[La^-]$
	LER	BLa = 0.0958 + 0.8196·HR + 0.1423·CBT + 0.2028·Forehead[LER]
Chest	$[La^-]sw$	BLa = 0.0315 + 0.9422·HR + 0.1332·CBT + 0.1032·Chest$[La^-]$
	LER	BLa = 0.0405 + 0.8652·HR + 0.0510·CBT + 0.0598·Chest[LER]
Back	$[La^-]sw$	BLa = 0.0381 + 0.9065·HR + 0.1572·CBT + 0.0829·Back$[La^-]$
	LER	BLa = 0.0394 + 0.8354·HR + 0.1572·CBT + 0.0829·Back[LER]

All regression models were developed using standardized (z-score transformed) variables. Therefore, the coefficients presented in the equations represent standardized regression coefficients (β), indicating the relative contribution of each predictor to BLa estimation. BLa, blood lactate concentration; HR, heart rate; CBT, core body temperature; $[La^-]sw$, sweat lactate concentration; LER, lactate excretion rate.

Supplementary Table S3. Fixed-effect estimates, standard errors, p-values, and AIC values for the final

Model	Predictor	Coefficient	SE	p-value	AIC
HR + CBT + Forehead [La$^-$]sw	HR	0.0231	0.001	<0.001	-7.234
HR + CBT + Forehead [La$^-$]sw	CBT	0.108	0.047	0.023	-7.234
HR + CBT + Forehead [La$^-$]sw	Forehead [La$^-$]sw	0.030	0.009	0.001	-7.234
HR + CBT + Chest [La$^-$]sw	HR	0.024	0.001	<0.001	5.199
HR + CBT + Chest [La$^-$]sw	CBT	0.093	0.049	0.060	5.199
HR + CBT + Chest [La$^-$]sw	Chest [La$^-$]sw	0.030	0.013	0.021	5.199
HR + CBT + Back [La$^-$]sw	HR	0.023	0.001	<0.001	7.876
HR + CBT + Back [La$^-$]sw	CBT	0.094	0.048	0.051	7.876
HR + CBT + Back [La$^-$]sw	Back [La$^-$]sw	0.031	0.016	0.053	7.876
HR + CBT + Forehead LER	HR	0.025	0.001	<0.001	-2.142
HR + CBT + Forehead LER	CBT	0.084	0.050	0.090	-2.142
HR + CBT + Forehead LER	Forehead LER	<0.000	0.001	0.120	-2.142
HR + CBT + Chest LER	HR	0.024	0.001	<0.001	9.382
HR + CBT + Chest LER	CBT	0.087	0.050	0.081	9.382
HR + CBT + Chest LER	Chest LER	0.002	0.001	0.278	9.382
HR + CBT + Back LER	HR	0.022	0.001	<0.001	5.109
HR + CBT + Back LER	CBT	0.098	0.048	0.041	5.109
HR + CBT + Back LER	Back LER	0.004	0.002	0.011	5.109

SE, standard error; AIC, Akaike information criterion; CBT, core body temperature; [La$^-$]sw, sweat lactate concentration; LER, lactate excretion rate. Coefficients, standard errors, and p-values are reported for the fixed effects of each final multivariable regression model. AIC is reported at the model level and is repeated across predictors within the same model for readability.

Supplementary Table S4. Assumption checks for candidate regression models.

Model	Shapiro–Wilk p	Breusch–Pagan p	Singular fit	Convergence warning
HR	0.022	0.668	No	No
HR+CBT	0.063	0.351	No	No
HR+Forehead[La⁻]sw	0.557	0.024	No	No
HR+Chest[La⁻]sw	0.473	0.661	No	No
HR+Back[La⁻]sw	0.159	0.669	No	No
HR+Forehead LER	0.244	0.036	No	No
HR+Chest LER	0.386	0.920	No	No
HR+Back LER	0.411	0.980	No	No
HR+CBT+Forehead[La⁻]sw	0.885	0.256	No	No
HR+CBT+Chest[La⁻]sw	0.692	0.740	No	No
HR+CBT+Back[La⁻]sw	0.209	0.549	No	No
HR+CBT+Forehead LER	0.465	0.467	No	No
HR+CBT+Chest LER	0.418	0.943	No	No
HR+CBT+Back LER	0.474	0.915	No	No
CBT	0.004	0.952	No	No
CBT+Forehead[La⁻] sw	0.177	0.260	No	No
CBT+Chest[La⁻]sw	0.010	0.033	No	No
CBT+Back[La⁻]sw	0.042	0.662	No	No
CBT+Forehead LER	0.963	0.565	No	No
CBT+Chest LER	0.027	0.272	No	No
CBT+Back LER	0.126	0.476	No	No

Note. Residual diagnostics were reviewed for all candidate models. No singular fit or convergence warnings were observed in any model. Lower p-values in the Shapiro–Wilk or Breusch–Pagan tests may indicate minor deviations from normality or homoscedasticity, respectively; these diagnostics were interpreted together with residual plots and Q–Q plots when evaluating model assumptions.
